# siRNA inhibition of telomerase enhances the anti-cancer effect of doxorubicin in breast cancer cells

**DOI:** 10.1186/1471-2407-9-133

**Published:** 2009-05-05

**Authors:** Xuejun Dong, Anding Liu, Cindy Zer, Jianguo Feng, Zhuan Zhen, Mingfeng Yang, Li Zhong

**Affiliations:** 1 The Molecular Medicine Center of Shaoxing People's Hospital, The First Affiliate Hospital of Shaoxing University, Shaoxing, 312000, PR China; 2 Division of Radiation Biology, Department of Cancer Biology and Lung Cancer and Thoracic Oncology Program, City of Hope and Beckman Research Institute, 1500 Duarte Road, Duarte, CA 91010, USA; 3 Cancer Hospital of Zhejiang Province, Hangzhou, 310022, PR China; 4 Department of Cell and Molecular Biology, Hebei University College of Life Sciences, 180 Wusi Road, Baoding, 071002, PR China

## Abstract

**Background:**

Doxorubicin is an effective breast cancer drug but is hampered by a severe, dose-dependent toxicity. Concomitant administration of doxorubicin and another cancer drug may be able to sensitize tumor cells to the cytotoxicity of doxorubicin and lowers the therapeutic dosage. In this study, we examined the combined effect of low-dose doxorubicin and siRNA inhibition of telomerase on breast cancer cells. We found that when used individually, both treatments were rapid and potent apoptosis inducers; and when the two treatments were combined, we observed an enhanced and sustained apoptosis induction in breast cancer cells.

**Methods:**

siRNA targeting the mRNA of the protein component of telomerase, the telomerase reverse transcriptase (hTERT), was transfected into two breast cancer cell lines. The siRNA inhibition was confirmed by RT-PCR and western blot on hTERT mRNA and protein levels, respectively, and by measuring the activity level of telomerase using the TRAP assay. The effect of the hTERT siRNA on the tumorigenicity of the breast cancer cells was also studied *in vivo *by injection of the siRNA-transfected breast cancer cells into nude mice.

The effects on cell viability, apoptosis and senescence of cells treated with hTERT siRNA, doxorubicin, and the combined treatment of doxorubicin and hTERT siRNA, were examined *in vitro *by MTT assay, FACS and SA-β-galactosidase staining.

**Results:**

The hTERT siRNA effectively knocked down the mRNA and protein levels of hTERT, and reduced the telomerase activity to 30% of the untreated control. *In vivo*, the tumors induced by the hTERT siRNA-transfected cells were of reduced sizes, indicating that the hTERT siRNA also reduced the tumorigenic potential of the breast cancer cells. The siRNA treatment reduced cell viability by 50% in breast cancer cells within two days after transfection, while 0.5 μM doxorubicin treatment had a comparable effect but with a slower kinetics. The combination of hTERT siRNA and 0.5 μM doxorubicin killed twice as many cancer cells, showing a cumulative effect of the two treatments.

**Conclusion:**

The study demonstrated the potential of telomerase inhibition as an effective treatment for breast cancer. When used in conjunction to doxorubicin, it could potentiate the cytotoxic effect of the drug to breast cancer cells.

## Background

Breast cancer is the most common cancer diagnosed in American women and is the second leading cause of cancer-related deaths [1]. About 200,000 new cases are diagnosed each year in the United States [2]. Chemotherapy is frequently used to relieve symptoms in advanced breast cancer patients and to reduce the risk of recurrence in patients with localized breast cancer. Doxorubicin (trade name Adriamycin) is one of the most commonly used drugs to treat breast cancer [1,3]. Monotherapy with doxorubicin has a good response rate of 10–50% [1], and doxorubicin-containing combination therapies usually result in better survival rate [4,5]. Other than for breast cancer treatment, doxorubicin is also used to treat a wide variety of solid tumors and hematological malignancies [6].

The clinical utility of doxorubicin and other anthracyclines are limited by their toxicity. Among the side effects are myelosuppression, acute nausea and vomiting, alopecia and cardiotoxicity related to cumulative dose [6,7]. Advances have been made in drug formulation and schedules of chemotherapy to better the safety profile and efficacy of doxorubicin. Liposomal doxorubicin formulations had been developed to counter the cardiotoxicity and increase the therapeutic index of the conventional anthracyclines [7]. Shortening the period between treatments in chemotherapy schedules also seems to increase the drug's effectiveness [1,2].

Doxorubicin induces single and double strand breaks in DNA mediated by topoisomerase II [8]. The ubiquitous expression of topoisomerases contributes to the non-selective targeting of doxorubicin, and is a major reason for its toxicity [6]. The toxicity of doxorubicin can be reduced if it is used in conjunction with another, more tumor-specific treatment in order to reduce the dosage. Telomerase plays a vital role in tumor proliferation. It synthesizes the telomeric repeats at the ends of chromosomes and replaces the progressively lost end sequences during each cell cycle, allowing cells to escape mortality and continue to proliferate. Telomerase is relatively specifically expressed in many tumor tissues, including breast cancer, and is repressed in most normal somatic tissues [9-12]. The tumor-specific expression of telomerase has made it a highly attractive cancer therapy target [13].

One method of specific inhibition of telomerase is through RNA interference (RNAi). Since its discovery, RNAi has been shown as a potent post-transcriptional gene silencing mechanism [14,15]. Introduction of small interfering RNAs (siRNAs) can mediate the specific degradation of the mRNA, whose sequence is contained in the siRNAs. The strong and specific suppression of gene expression by RNAi is currently being evaluated as a potentially useful method for developing gene-silencing therapies for cancer [16,17]. There have been reports which demonstrated that RNAi against telomerase reverse transcriptase (hTERT), the protein component of telomerase, could successfully inhibit telomerase activity in several cancer cell lines. [18-23].

In this study, we hypothesized that a combination treatment of doxorubicin and hTERT siRNA could sensitize tumor cells to doxorubicin and thus enhance its cytotoxicity. We first characterized the effect of our hTERT siRNA in promoting breast cancer cells apoptosis *in vitro *and *in vivo*. We then compared the effects of hTERT siRNA, low dose doxorubicin and the combined treatment of the two to see if a cumulative effect would be observed when the two treatments were combined.

## Methods

### siRNA preparation

The siRNA sequences were designed by a commercial software (Applied Biosystems/Ambion, Austin, TX). For hTERT, the siRNA sense sequence is 5'-UGAUUUCUUGUUGGUGACAdTdT-3', and the anti-sense sequence is 5'-UGUCACCAACAAGAAAUCAdTdT-3'. The negative control siRNA sense sequence is 5'-GGCCUCAGCUGCGCGACGCdTdT-3', and the antisense sequence is 5'-GCGUCGCGCAGCUGGGCCAdTdT-3'. The selected sequences were submitted to BLAST http://www.ncbi.nlm.nih.gov/blast/ to ensure that the selected gene was targeted specifically. The siRNAs were synthesized and sequenced by Guangzhou RiboBio Co., Ltd, Ghuangzhou, China.

### Cell culture

The breast cancer cell lines, MCF-7 and MDA-MB-453 (both purchased from American Type Culture Collection, Manassas, VA), were grown in Dulbecco's Modified Eagle's Medium (DMEM) supplemented with 10% fetal bovine serum, 100 U/ml penicillin and 100 μg/ml streptomycin at 37°C and 5% CO_2_. All culture medium and supplements were purchased from Invitrogen, Carlsbad, CA.

### hTERT siRNA transfection

MCF-7 cells (2 × 10^5 ^cells per well) and MDA-MB-453 cells (3 × 10^5 ^cells per well) were plated in 6-well plates and allowed to grow overnight. 200 pmol siRNA and 5 μl Lipofectamine™ 2000 (Invitrogen, Carlsbad, CA) were diluted in OPTI-MEM (Invitrogen, Carlsbad, CA) to a total volume of 250 μl. The diluted siRNA and Lipofectamine™2000 were mixed and incubated at ambient temperature for 20 minutes. The cells were washed with serum-free DMEM medium, and then the diluted siRNA mix was added to the 6-well plates for 6 hours, after which the mix was replaced with growth medium.

### Reverse transcription and quantitative PCR (RT-qPCR)

Cells were collected 48 hours after siRNA transfection. Total RNA was isolated by TRIzol Reagent (Invitrogen, Carlsbad, CA) using the manufacturer's single-step chloroform-extraction protocol. Complementary DNA was synthesized using the First-strand cDNA synthesis kit (Takara, Shiga, Japan). An aliquot of 1 μg of total RNA was reverse-transcribed by MMLV transcriptase (Invitrogen, Carlsbad, CA) using random hexamer primers according to the manufacturer's instructions. The reaction system of real-time PCR with dual-labeled Taqman probes was: 10 μl of 2 × Premix EX Tag TM buffer, 0.4 μl of 10 pmol/ml of each primer, 0.8 μl of probe, 2 μl cDNA and 6.4 μl nuclease-free distilled water. For hTERT, the sense primer sequence is 5'-CCGTCTGCGTGAGGAGATC-3', and the anti-sense primer sequence is 5'-TCCGGTAGAAAAAGAGCCTGTT-3', and the Taqman probe 5'-FAM-GGCCAAGTTCCTGCACTGGCTGACT-TAMRA-3'. For GAPDH, the sense primer sequence is 5'-CCAGGTGGTCTCCTCTGACTT-3', and the anti-sense primer sequence is 5'-GTTGCTGTAGCCAAATTCGTTGT-3', and the Taqman probe 5'-FAM-AACAGCGACACCCACTCCTCCACC-Eclipse-3'. Reaction parameters were: 95°C for 10 s, then 95°C for 5 s and 60°C for 20 s, 40 cycles. Relative gene expression of hTERT was calculated with the 2^-(ΔΔCT) ^method [24], using GAPDH as the reference gene.

### Detection of hTERT protein expression

Cells were collected 48 hours after siRNA transfection. After washing with pre-chilled phosphate-buffered saline (PBS), cells were lysed in 1 ml of 1% Nonide P-40, 25 mM Tris-HCl, 150 mM NaCl, 10 mM EDTA, pH8.0, containing a 1:50 dilution of a protease inhibitor mixture for 30 min on ice. hTERT protein was separated using a 5% sodium dodecyl sulfate-polyacrylamide gel electrophoresis (SDS-PAGE). The β-actin loading control was separated using a 10% SDS-PAGE. Proteins were electrotransferred onto a nitrocellulose membrane and blocked for 3 hours in 3% bovine serum albumin after the transfer. Membranes were incubated overnight at 4°C with an hTERT primary antibody (Santa Cruz Biotechnology, Santa Cruz, CA), then washed and incubated with an alkaline phosphatase-conjugated secondary antibody (Santa Cruz Biotechnology, Santa Cruz, CA) in TBST for 2 hours. The bands were visualized using NBT/BCIP color substrate and were scanned and analyzed.

### Telomerase activity assay

Telomerase activity was determined by using a PCR-based telomeric repeat amplification protocol (TRAP) enzyme-linked immunosorbent assay (ELISA) kit (Roche, Mannheim, Germany) according to manufacturer's protocol. In brief, MCF-7 and MDA-MB-453 cells were collected 48 hours after the siRNA transfection. The cells were washed three times with cold PBS, homogenized in 200 μl cell lysis buffer, and incubated on ice for 30 min. For the TRAP reaction, 2 μl of cell extract was added to 25 μl of reaction mixture and sterile water was added to a final volume of 50 μl. PCR was then performed as follows: prime elongation (20 min, 25°C), telomerase inactivation (5 min, 94°C), product amplification for 30 cycles (94°C for 30 s, 50°C for 30 s, 72°C for 90 s) and then balance (10 min at 72°C). 5 μl of PCR products were bound to a streptavidin-coated 96 well plate and hybridized to a digoxigenin (DIG)-labeled telomeric repeat specific detection probe. The immobilized PCR products were detected with peroxidise conjugated anti-DIG antibody. After addition of the stop reagent, the plate was assessed on a plate reader at a wavelength of 450 nm within 30 min.

### MTT assay for cell viability

MCF-7 and MDA-MB-453 cells (2 × 10^4 ^cells/well for both cell lines) were incubated in 96-well plates each contained 200 μl of medium. The cells were divided into six groups: 1) blank group; 2) control siRNA group; 3) hTERT siRNA group; 4) blank and doxorubicin group; 5) control siRNA and doxorubicin group; 6) hTERT siRNA and doxorubicin group. Transfection of siRNAs was done the following day as described previously. 12 hours after the siRNA transfection, the cells of the appropriate groups were treated with doxorubicin (Zhejiang Hisun Pharmaceutical Co, Ltd, Taizhou, China) to a final concentration of 0.5 μM. The rate of cellular proliferation was measured every 24 hours for 96 hours. At the end of each time point, 20 μl of 5 mg/ml MTT (Sigma, St. Louis, MO) was added to each well. Four hours later, 200 μl of DMSO was added to the MTT-treated wells and the absorption at 492 nm was determined on a spectrometer. Each experimental condition was carried out in triplicate.

### Flow cytometry for cell apoptosis detection

MCF-7 cells and MDA-MB-453 cells were plated in 6-well plates and were divided into the same six groups as in the MTT assay. The siRNA transfection and doxorubicin treatment were as before. After 48 hours, cells were collected and washed twice with pre-chilled PBS. The cell concentration was adjusted to 5 × 10^5^~5 × 10^6 ^cells/well with 100 μl of pre-chilled binding buffer. 5 μl of Annexin V-FITC and 5 μl of propidium iodide (PI) were added to each sample and incubated for 10 min in the dark on ice. 400 μl of pre-chilled binding buffer was added at the end. The cell apoptosis was determined by flow cytometry. Apoptosis of each group was assayed three times.

### Senescence associated β-galactosidase staining

MCF-7 cells and MDA-MB-453 cells were divided into the same six groups as in the MTT assay. The siRNA transfection and doxorubicin treatment were as before. After 48 hours, cells were rinsed three times with PBS and fixed in 2% formaldehyde, 0.2% glutaraldehyde for 5 minutes. The cells were washed again with PBS and stained overnight at 37°C in β-galactosidase stain solution containing 1 mg/ml 5-bromo-4-chloro-3-inolyl-β-galactosidase, 40 mM citric acid/sodium phosphate (pH = 6.0), 5 mM potassium ferrocyanide, 150 mM NaCl, and 2 mM MgCl_2_. After staining, the cells were rinsed with PBS and the stained cells were observed under a microscope. The percentage of β-galactosidase-positive cells was calculated by counting 3 representative fields of at least 100 cells in each group.

### Tumor induction in nude mice with breast cancer cells injection

The animal study was approved by the First Affiliate Hospital of Shaoxing University (Shaoxing, China). Female BALB/c nude (nu/nu) mice were purchased from SLAC Laboratory Animals (Shanghai, China) and were divided into three groups with 5 mice in each group: the untreated group, the negative siRNA group, and the hTERT siRNA group. Six hours after siRNA transfection, cells were trypsinized, washed with PBS, and resuspended in serum-free DMEM to a concentration of 2 × 10^7 ^cells/ml. 2 × 10^6 ^cells (i.e., 0.1 ml of the resuspended cells) were injected into each flank of the mice subcutaneously. The tumor volumes were measured with a caliper every 3 days for 30 days. Tumor volume was calculated by the formula of tumor volume (mm^3^) = (d^2 ^× D)/2, where d is the short diameter, and D is the long diameter. All measurements were performed in a coded, blinded fashion. The experiment was done with both MCF-7 and MDA-MB-453 cells.

## Results

### 1. Down-regulation of telomerase activity in breast cancer cells after siRNA transfection

We initially tested three sets of siRNAs for the hTERT knockdown. Preliminary results showed one set being particular effective (data not shown); thus, all experiments described in this report were conducted with this siRNA (sequence see methods section).

**Figure 1:**
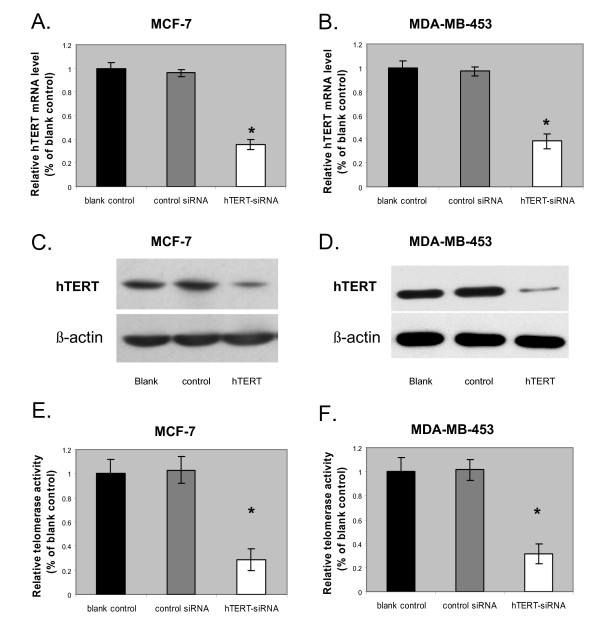
**Effects of hTERT-siRNA knock-down in breast cancer cells**. (A and B) hTERT mRNA expression levels quantified by RT-PCR in MCF-7 (A) and MDA-MB-453 (B) cells at 48 h after a 6 h exposure in hTERT siRNA, control siRNA, or untransfected, respectively. Relative quantification of hTERT mRNA expression levels was accomplished by the Pfaffl method of  Data are shown as mean ± SD (error bar) of 3 experiments; *, p = 0.001, two-tailed student's t-test. (C and D) representative western blots showing the expression of hTERT protein expression levels at 48 h after a 6 h exposure in hTERT siRNA (right lane; hTERT), control siRNA (middle lane; control), or untransfected (left lane; blank), respectively, in MCF-7 (C) and MDA-MB-453 (D) cells. β-actin was the internal loading control. (E and F) Telomerase activity levels quantified by TRAP assay in MCF-7 (E) and MDA-MB-453 (F) cells at 48 h after a 6 h exposure in hTERT siRNA, control siRNA, or untransfected, respectively. Data are shown as mean ± SD (error bar) of 3 experiments; *, p = 0.001, two-tailed student's t-test.

The hTERT siRNA was transiently transfected into the breast cancer cell lines MCF-7 and MDA-MB-453. After 48 hours, the hTERT mRNA and protein levels were quantified by real time RT-PCR and western blots, respectively. As shown in Fig. 1A and 1B, hTERT siRNA transfection significantly reduced the amount of hTERT mRNA. The level of hTERT mRNA in the hTERT siRNA-treated group was about 40% of the blank group in MCF -7 cells and about 38% in MDA-MB-453 cells. The control siRNA had no effect on the hTERT mRNA level in either cell line. The results between MCF-7 and MDA-MB-453 cells were not significantly different, indicating that the effect of the hTERT siRNA was specific. The hTERT siRNA was also successful in knocking down hTERT protein expression. As shown in Fig. 1C and 1D, while the β-actin internal control showed equal loading among the three groups, the level of hTERT protein was noticeably lower in the hTERT siRNA-treated group compared to both the blank and the negative siRNA-treated groups, suggesting that the hTERT siRNA treatment could effectively reduce the hTERT protein level.

The level of telomerase activity in human breast cancer has been shown to significantly correlate with that of the hTERT mRNA expression [25]. Consistent with this, we found that the hTERT siRNA transfected cells showed a 70% reduction in telomerase activity, as determined by a PCR-based telomeric repeat amplification protocol (TRAP) ELISA (Fig. 1E and 1F)

### 2. hTERT siRNA inhibited cell viability in vitro and in vivo

**Figure 2:**
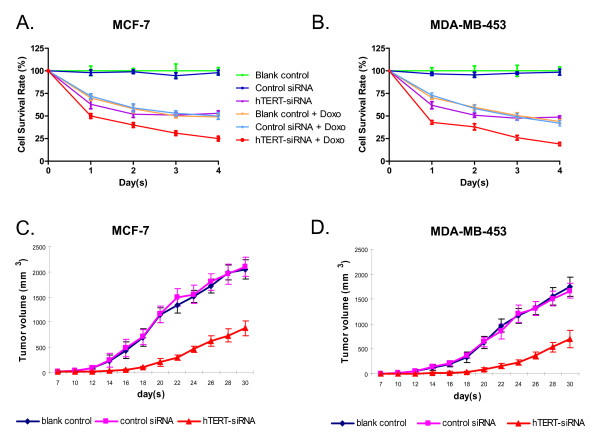
**Effects of hTERT-siRNA combined with doxorubicin on the proliferation of breast cancer cells**. The MCF-7 (A) and MDA-MB-453 (B) breast cancer cells were either untreated or treated with 0.5 μM doxorubicin 12 h after the siRNA transfection. Cell viability was measured by the MTT assay every day for 4 days. Data are shown as mean ± SD from three independent experiments. Panel C and D show the inhibition effect of hTERT siRNA on the tumorigenic potential of human breast cancer cells. 2 × 10^6 ^MCF-7 cells (C) or MDA-MB-453 cells (D) that were untreated or transfected with control siRNA or hTERT siRNA were injected subcutaneously into each flank of athymic nude mice. The tumor dimensions were measured every 3 days. The mean tumor volume (mm^3^) was calculated according to the formula: (d^2 ^× D)/2, where d and D are the shortest and longest diameters of the tumor, respectively. All measurements were performed in a coded, blinded fashion. Data are shown as mean ± SD with 5 mice per treatment group.

Decreased telomerase activity is associated with arrested cell growth; we thus sought to determine whether or not the hTERT siRNA-induced reduction in telomerase activity would affect cell viability in the two breast cancer cell lines. The cells were transfected with the hTERT siRNA as previously, and the amount of viable cells was determined by the MTT assay every 24 hours for four days. As shown in Fig. 2A and 2B, the hTERT siRNA significantly decreased the percentage of viable cells in both cell lines. The effect of the siRNA was similar in both cell lines. The decrease was rapid: only ~63% of cells were alive after 24 hours, and only 50% of cells survived after 48 hours. The number of surviving cells remained at about 50% from there on and seemed to be heading into a recovery trend, consistent with the transient nature of the siRNA transfection.

We next determined whether or not the hTERT siRNA could negatively affect the *in vivo *growth of the breast cancer cells. We tested the ability of the hTERT siRNA to down-regulate the tumorigenic potential of the two cancer cells lines. Cells that were transfected with hTERT siRNA, control siRNA or untransfected cells were injected into the flanks of nude mice, and the volume of the tumors induced were measured every three days for thirty days. As shown in Fig. 2C and 2D, although all three groups of mice developed tumors, the growth of the tumors from the hTERT siRNA-transfected cells was significantly slower, and the volume of tumors was much smaller, as compared to the groups that received the untransfected and the control siRNA-transfected cells. At the end of the thirty days, the hTERT-siRNA transfected tumors were, on average, about 40% the volume of the two control groups. The volumes of tumor between the two control groups were not different, showing the specificity of the effect of the hTERT siRNA.

### 3. Decrease in cell viability caused by the hTERT siRNA was due to increase in apoptosis

**Figure 3:**
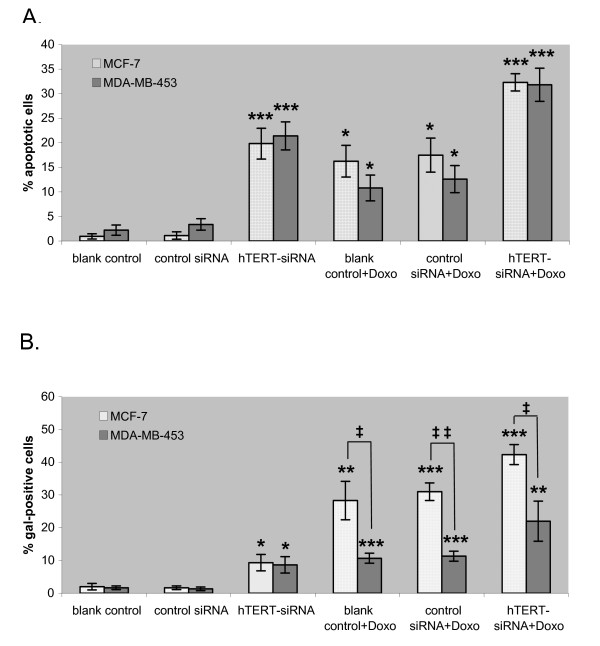
**Effect of hTERT-siRNA combined with doxorubicin on apoptosis and senescence of breast cancer cell lines**. (A) Apoptosis of breast cancer cells induced by hTERT-siRNA and doxorubicin. MCF-7 and MDA-MB-453 cells were untreated (blank control), transfected with control or hTERT siRNA, and then treated with 0.5 μM doxorubicin. After 48 h of incubation, apoptotic cells were detected by FACS. Percentages of apoptotic cells are shown as mean of 3 experiments ± SD (error bar); *, p < 0.01; ***, p < 0.0001, two-tailed student's t-test. (B) Senescence of breast cancer cells induced by hTERT-siRNA and doxorubicin. MCF-7 and MDA-MB-453 cells were untreated (blank control), transfected with control or hTERT siRNA, and then treated with 0.5 μM doxorubicin. After 48 h of incubation, cells were stained for β-galactosidase activity. The percentage of β-galactosidase-positive cells was calculated by counting 3 representative fields of at least 100 cells in each group, shown as the mean ± SD (error bar); *, p < 0.01; **, p < 0.005; ***, p < 0.0001; ‡, p < 0.01 between cell types; ‡‡, p < 0.001 between cell types.

To determine whether the decrease in cell viability caused by the hTERT siRNA was due to an increase in apoptosis or a decrease in cell proliferation, we checked the amount of apoptotic cells from the untransfected, control siRNA-transfected and hTERT siRNA-transfected cells by Annexin V-FITC and propidium iodide (PI) labeling followed by fluorescence-activated cell sorting (FACS). 48 hours after the siRNA transfection, both cell lines had about 20% of the hTERT siRNA-transfected cells as apoptotic, i.e., Annexin V-FITC and/or PI positive, while cells in the blank and the control siRNA groups were almost all non-apoptotic (Fig. 3A).

### 4. hTERT siRNA and doxorubicin treatments combined could additively increased breast cancer cell apoptosis

We also determined the cytotoxicity of low-dose doxorubicin alone on our cell lines. We treated both cell lines with 0.5 μM doxorubicin and carried out the MTT assay and FACS for apoptotic cells as were for the siRNA treated cells. The doxorubicin concentration is low when compared to the actual plasma concentration of free doxorubicin, which could reach 50 μM [26]. The doxorubicin treatment had a cytotoxicity comparable to the hTERT-siRNA treatment, but with a somewhat slower kinetics and did not plateau by day 4 (Fig. 2A and 2B). The FACS analysis showed that the MCF-7 cells were equally prone to apoptosis induction by either treatment. The MDA-MB-453 cells seemed to have a higher resistance to doxorubicin-induced apoptosis than siRNA-induced apoptosis, although the difference did not reach statistical significance (Fig. 3A).

The combined use of doxorubicin and hTERT-siRNA showed a cumulative effect. The cells were first transfected with the siRNA, and then doxorubicin was added. From the MTT assay, already half or more than half of the cells did not survive by the end of the first day of the treatments. The decline in viable cells then followed the rate of decline of doxorubicin treatment alone. By the end of day 4, more than twice as many cells were dead from the combined treatment (Fig. 2). Both cell lines behaved similarly towards the treatments. The FACS analysis revealed that about 32% of the cells for both cell lines were apoptotic, which was roughly close to the sum of the apoptotic cells caused by hTERT siRNA and doxorubicin alone, thus again showing an additive effect of the combined treatment.

### 5. The breast cancer cell lines showed differential senescence response to doxorubicin

Telomeric dysfunctions can trigger cells to enter replicative senescence [27,28], a mechanism by which tumor cells can lose their replicative capacity by entering a terminally arrested state. We therefore examined if the hTERT siRNA transfection would induce cells to enter senescence. We found that only about 10% of the hTERT siRNA transfected cells of both cell lines were β-galactosidase positive (Fig. 3B). Thus, our siRNA treatment most likely did not "activate" senescence.

Doxorubicin has been known to cause cell to senesce [27-30]. We examined the senescence of the two cell lines under our doxorubicin treatment conditions. Interestingly, we found that our two cell lines reacted differently towards doxorubicin in terms of senescence. As expected, MCF-7 cells showed considerable senescence (about one-third of cells; Fig. 3B, white bars). On the other hand, only about 10% of MDA-MB-453 cells were β-galactosidase positive (Fig. 3B, grey bars). This was in contrast to the similar apoptotic response of the two cell lines towards doxorubicin. Finally, the combined treatment of hTERT siRNA and doxorubicin also elicited a differential senescence response: significantly fewer of the MDA-MB-453 cells showed senescence than did the MCF-7 cells, but both cells lines underwent apoptosis similarly (Fig. 3A and 3B).

## Discussion

There have been consistent efforts in enhancing the effect of doxorubicin treatment in human cancer cells through the concerted inhibition of telomerase [19,31-33]. Our present study investigated whether the direct addition of hTERT siRNA as the method of telomerase inhibition would enhance the cytotoxicity of low dose doxorubicin on breast cancer cells. We showed that the combined treatment of hTERT siRNA and doxorubicin indeed caused more breast cancer cells deaths than either treatment alone.

The effectiveness of siRNA inhibition is highly sequence-dependent. We were able to find one which had a robust effect. Unlike small molecule inhibitors of hTERT [34,35], or methods that target the RNA component of telomerase [36,37], where a long lag time is observed due to telomere attrition before apoptosis from senescence can occur, the apoptosis induction by our siRNA transfection was relatively rapid. We speculated at first that the rapid response was a result of telomere uncapping. The telomeres are capped by telomerase and its associated proteins which physically protect the telomeric ends. Disruption of the cap due to the reduced hTERT level may trigger a p53-independent, telomere-associated DNA-damage apoptosis [10,11]. However, Gandellini et al. [18] have shown that the rapid inhibition by their hTERT siRNA was not due to telomere uncapping or shortening. It would be interesting to know if the rapid apoptosis induction by siRNA is p53 dependent, and if any of the DNA repair pathways may be involved. Recent studies on telomerase have also shown that telomerase is involved in DNA repair and is a binding partner to proteins that function in cell viability and proliferation [38,39]. Disruption of hTERT expression thus may be impairing other vital cellular functions as well, which may be another reason for the robustness of the hTERT siRNA in inducing apoptosis.

Our results showed a rapid apoptosis induction by both the hTERT siRNA and doxorubicin, but a cell type dependent senescence response, which is in disagreement with published results, where doxorubicin did not induce apoptosis, but rather senescence in MCF-7 cells [28]. Discrepancies among the same cell line arisen from culture may certainly lead to different responses, and the dose schedule and end point selection may also explain the differences. Our cells were continuously exposured to 0.5 μM doxorubicin for 2–4 days, while Elmore et al.'s study [28] exposed the cells to 1 μM doxorubicin for 2 hours, and assayed 5 days later. It was also interesting to see our two cell lines senesced differently towards doxorubicin. Presumably, our MDA-MB-453 cells and MCF-7 cells have wild type p53 activity. MDA-MB-453 cells are, however, estrogen receptor alpha negative. Further investigation into these differences will most certainly add to our understanding of the mechanisms controlling cell fate.

A very low dose of doxorubicin (0.5 μM) was used in our studies. The actual plasma concentration of free doxorubicin that is reached upon intravenous bolus administrations into average sized persons can reach 50 μM [26]. We are now determining if the same cumulative effect can still be seen in a doxorubicin-resistant breast cancer cell line and if it can be done at an even lower dose of doxorubicin as well.

The *in vivo *tumor growth experiment clearly demonstrated impaired growth of the tumors formed by the hTERT siRNA transfected MCF-7 cells. While admittedly the experiment did not directly address the ability of hTERT siRNA to induce apoptosis in pre-existing tumors, the results nonetheless reflect the possible beneficiary effects of siRNA therapies and validates ongoing efforts in optimizing a siRNA delivery system into tumors. Our *in vitro *results also suggested that a combination treatment of breast cancer cells with siRNA together with doxorubicin can represent a new approach of breast cancer treatment. The combined treatment of hTERT siRNA and doxorubicin almost doubled the number of apoptotic cells than when either treatment was used alone. Our ultimate goal will be to extend our *in vitro *findings on the combined effects of hTERT siRNA and doxorubicin in promoting breast cancer cells apoptosis *in vivo*. The technology of siRNA delivery is rapidly developing. Aside from the traditional, viral-based delivery systems [17], nanotechnology is also being applied [40,41]. As mentioned before, liposomal doxorubicin is already an approved treatment for breast cancer; albumin nanoparticulate chaperones of paclitaxel were approved for locally recurrent and metastatic breast cancer in 2005 [7,42,43]. It is foreseeable that a simultaneous delivery of doxorubicin and siRNA may become feasible with a nanoparticle encapsulation system.

## Conclusion

The study demonstrated the potential of inhibiting telomerase as an effective treatment of breast cancer when used alone and, when used in conjunction to doxorubicin, could potentiate the cytotoxic effect of the drug to breast cancer cells.

## Abbreviations

FACS: fluorescence-activated cell sorting; FAM: 6-carboxyfluorescein; FITC: fluorescein isothiocyanate; hTERT: human telomerase reverse transcriptase; MTT: 3-(4, 5-dimethylthiazol-2-yl)-2, 5-diphenyltetrazolium bromide; PI: propidium iodide; RNAi: RNA interference; siRNA: small interfering RNA; TAMRA: 6-carboxy-tetramethyl-rhodamine; TRAP: telomeric repeat amplification protocol.

## Competing interests

The authors declare that they have no competing interests.

## Authors' contributions

XD was involved in the experimental design, data interpretation and manuscript revision. AL carried out most of the experiment and data analysis. CZ was in charge of the data analysis and manuscript preparation. JGF did the animal model study. ZZ and MY were involved in protein analysis. LZ made contributions to data interpretation and manuscript preparation.

## Pre-publication history

The pre-publication history for this paper can be accessed here:

http://www.biomedcentral.com/1471-2407/9/133/prepub
